# Automatic dispensing cabinets and governance of controlled drugs: an exploratory study in an intensive care unit

**DOI:** 10.1136/ejhpharm-2020-002552

**Published:** 2021-05-11

**Authors:** Valentina Lichtner, Mirela Prgomet, Peter Gates, Bryony Dean Franklin

**Affiliations:** 1 Department of Management, Leeds University Business School, University of Leeds, Leeds, UK; 2 Centre for Health Systems and Safety Research (CHSSR), Australian Institute of Health Innovation, Macquarie University, Sydney, New South Wales, Australia; 3 Department of Practice and Policy, UCL School of Pharmacy, University College London, London, UK; 4 Centre for Medication Safety and Service Quality, Pharmacy Department, Imperial College Healthcare NHS Trust, London, UK

**Keywords:** hospital distribution systems, medical informatics, medication systems, hospital, pharmacy service, hospital, health services administration

## Abstract

**Background:**

Governance of controlled drugs (CDs) in hospitals is resource intensive but important for patient safety and policy compliance.

**Objectives:**

To explore whether and how storing CDs in an automated dispensing cabinet (ADC) in a children’s hospital intensive care unit (ICU) contributes to the effectiveness and efficiency of CD governance.

**Methods:**

We conducted a mixed-methods exploratory study, comprising observations, interviews and audits, 3 months after ADC implementation. We observed 54 hours of medications activities in the ICU medication room (with 42 hours of timed data); interviewed nurses (n=19), management (n=1) and pharmacy staff (n=3); reviewed 6 months of ICU incident reports pertaining to CD governance; audited 6 months of CD register data and extracted logs of all ADC transactions for the 3 months following implementation. Data analysis focused on four main CD governance activities: safekeeping/controlling access, documenting use, monitoring, and reporting/investigating.

**Results:**

Nurses and pharmacists perceived spending less time on CD governance tasks with the ADC. The ADC supported CD governance through automated documentation of CD transactions; ‘blind counts’; automated count discrepancy checks; electronic alerts and reporting functionalities. It changed quality and distribution of governance tasks, such as removing the requirement for ‘nurses with keys’ to access CDs, and allowing pharmacists to generate reports remotely, rather than reviewing registers on the ward. For CDs in the ADC, auditing and monitoring appeared to be ongoing rather than periodic. Such changes appeared to create positive reinforcing loops. However, the ADC also created challenges for CD governance. Most importantly, it was not suitable for all CDs, leading to workarounds and parallel use of a safe plus paper registers.

**Conclusions:**

ADCs can significantly alter CDs governance in clinical areas. Effects of an ADC on efficiency and effectiveness of governance tasks appear to be complex, going beyond simple time savings or more stringent controls.

## Background

Effective governance of controlled drugs (CDs) in hospitals is necessary for the safety of staff and patients,^1–3^ compliance with the law^4–6^ and CD stewardship.^7^ We define CD governance in hospitals as the structures and processes to organise, secure, monitor and account for, supply and use of CDs. CD governance may involve trade-offs^8^ (such as making CDs more secure but less accessible in emergencies), and may result in limitations, such as error-prone, time-consuming record keeping via paper-based systems.^9 10^ CD governance may particularly affect clinical areas with high use of CDs, such as intensive care units (ICUs).^11^

Automated dispensing cabinets (ADCs) have been introduced in hospital wards to make CD governance more effective and efficient.^10 12 13^ However, evidence on the effects of ADCs on CD governance is limited, as most research on ADCs implementations is not focused specifically on CDs or their impact on CD governance.^14–17^ A few studies suggest that ADCs save nurses or pharmacists’ time on CDs governance, for example, due to the elimination of (some or all of) the daily counts of CDs, or because of less time spent updating paper registers.^10 18 19^ Reports of initiatives to identify CD diversion in US hospitals^20 21^ show how ADC may contribute to monitoring and investigations. However, the literature also suggests that issues remain with CD governance after ADC implementations.^22^

We aimed to explore whether and how an ADC contributes to the effectiveness and efficiency of governance of CDs and through what mechanisms.

## Methods

We carried out an exploratory, mixed-methods study of an ADC in a paediatric ICU, with a focus on CDs.

### The setting

The study was conducted in a children’s hospital in Sydney, Australia. The hospital implemented an ADC (Omnicell^23^) in the ICU on 26 March 2019.

### Study design

We applied both quantitative methods (structured observations, audits of registers and data extracted from ADC) and qualitative methods (unstructured observations, interviews, reviews of incident reports). The audits were able to capture CD governance data before and after ADC implementation retrospectively, while the other methods were limited to a post-implementation period. We considered medications as CDs if they were treated as such on the ICU concerned for the purpose of governance.

### Observations

Observations, structured and unstructured, were carried out by one researcher (VL) in the ICU medication room about 3 months after ADC implementation (June–July 2019). All activities carried out in the medication room were included. Structured observations were recorded using the Work Observation Method By Activity Timing (WOMBAT 3.0^24^) tool, which automatically timestamped recorded data. Unstructured observations were captured as field notes. ADC training sessions, where nurses received training as ‘super users’, were also observed.

### Interviews

Qualitative semi-structured interviews were conducted by one researcher (VL) with ICU nurses to gather their perceptions and experiences with using the ADC. All nurses present on the ward on the interview days were invited to participate and could do so individually or as a group. We also sought to interview pharmacy staff, implementation team and hospital management for their views. Sampling was purposive. With consent, interviews were audio-recorded and transcribed; otherwise, the researcher took concurrent field notes.

### Incidents

We (VL, PG) carried out a retrospective review of patient safety incidents voluntarily reported through the hospital incident reporting system. All ICU incident reports relating to medications for a period of about 3 months before and 3 months after ADC implementation (January–June 2019) were reviewed.

### Audits of controlled drugs registers and ADC transaction logs

Retrospective audits of entries documented in ICU CD paper registers, about 3 months before and 3 months after ADC implementation (January–June 2019) were carried out by two researchers (VL, PG). As a control, we also audited registers from the oncology and orthopaedics wards.

Data extracted from the registers included: date and time; drug name, strength, formulation; amounts (received, given, discarded, balanced); discrepancies (box 1) identified by staff as annotated in registers and number of asterisks to signal amendments to records. We also captured missing amounts. For the denominator, we recorded the number of CD doses administered or discarded.

Box 1 **Definition of controlled drug discrepancy**

A discrepancy is a difference between a medication balance in the register and the physical balance of that medication in stock in the ward.

Discrepancies with liquid medications in bottles may also be associated with the unaccounted space in syringes (‘dead space’). Each syringe would take approximately 0.2–0.4 mL of liquid extra to the intended dose. Over a number of doses taken from the same bottle of medication, this loss from ‘dead space’ can accumulate to a considerable amount and generate a discrepancy between the balance documented in the register and the actual volume of liquid in the bottle.

Other causes of discrepancies include calculation errors, missing data (eg, a spill not documented), wrong counts of remaining stock and potential diversions of medications.

Discrepancies can be either losses (less medication in stock than the recorded balance) or gains (more in stock than the recorded balance).

Two researchers (MP, VL) conducted a retrospective audit of all transactions recorded by the ADC for 3 months following implementation (26 March–30 June 2019). Data extracted included: date and time; drug name, strength, formulation; transaction types (issue, return, waste, cycle count); amounts (issued, discarded, counted, returned) and discrepancies by type (eg, cycle count discrepancy).

### Data analysis

The quantitative data analysis was conducted using descriptive statistics in SPSS (V.23)^25^ for the WOMBAT data and Excel for the audit data. For the CD register data, we assessed number and frequency of CD discrepancies, calculation errors and other inaccuracies in documentation. The analysis was performed per ward, per type of medication (single doses vs multidose bottles), before and after ADC implementation and as a comparison across wards. For the ADC transaction logs, we calculated the number of ADC-generated discrepancies and time to resolution of discrepancies. For all transactions of CDs removed from the ADC, we matched the corresponding ‘partial waste’ data and calculated time to waste (the time from removing a medication dose from the ADC and documenting partial waste). For the WOMBAT data, we mapped the corresponding ADC transaction log data to obtain details about the type of medication (CD or non-CD) involved in the recorded activities and calculated frequency and duration of activities for each medication type.

Qualitative data from field notes and interview transcripts were first coded inductively by one researcher (VL) using NVivo (V.12)^26^ to capture aspects related to systems (eg, ADC, safe and registries), medications (CDs and non-CDs), activities and participants’ views as well as the wider context of ICU medication work. Incidents were also analysed thematically in a spreadsheet. Initial analysis was then independently verified by two researchers (MP and PG), with disagreements discussed and resolved via consensus. We then focused the analysis on CD governance. We identified four main activities of CD governance (table 1) in the data and local/national policy^6^ and used this as a framework to analyse mechanisms supporting or hindering CD governance, integrating qualitative and quantitative data within this framework.

**Table 1:** Activities for the governance of controlled drugs (CDs) in hospital wards

CD governance activity	Description
Safekeeping/Controlling access	CDs (including those owned by patients) stored separately from other medications, in a cabinet or metal safe, kept locked when not in use. Access to CD cabinets’ keys strictly controlled. Access to CDs restricted to staff members authorised to administer them. Accesses witnessed by a staff member. Expired, unusable or unwanted CDs stored in the safe pending destruction.
Documenting transactions	Records kept of CD transactions in a drug register. Records of transactions signed by two staff members present during the activity (eg, nurse and witness). Transactions recorded in the CD register included: removal and replacing of CDs from the storage unit; discarding any unused portion of the CD and destruction of CDs.
Monitoring	Audits of CDs in stock performed to confirm records are meeting policy requirements and to detect any possible misappropriation. The recorded CDs balance checked daily against the physical balance in the cabinet and safe, by a registered nurse/midwife with a witness and documented in the register. Expired, unusable or unwanted CDs, pending destruction, are included in the routine stock checks.
Reporting and investigating	CDs discrepancies recorded and reported to the hospital management and authorities. Investigations conducted to explain the discrepancies.

## Results

Table 2 provides details of the data included in the study. We structure the findings around the main governance activities described in table 1. The focus is on the use of ADC for these activities, compared with a safe plus registers when appropriate. Table 3 provides a summary of the findings.

**Table 2:** Overview of methods and data type

Methods and data type	Description
Ethnographic observations; field notes	About 54 hours of observations were completed over 14 days in the period 6 June–24 July 2019. Periods of observations were of 2 hours 30 min on average (min 1 hour to max 7 hours). Three one-to-one sessions of nurse ADC super-user training were also observed.
Timed observations with WOMBAT; structured tasks and time stamps in spreadsheet	About 42 hours of observations were timed using the WOMBAT tool. Timed observations took place over 13 days between 6 June and 24 July 2019, average duration of individual observation session: 2 hours 23 min (range 1–47 hours).
Interviews; transcripts of audio recordings and field notes	Two 2-hour ‘drop-in sessions’ were organised to facilitate interviews with ICU nurses: 19 nurses participated. Interviews took place with ADC back-office pharmacist, ADC trainer, Deputy Director of Pharmacy, Clinical Program Director.
Incidents reports; structured and free text data in spreadsheet	We received a total of 171 incidents that had been reported in ICU in relation to medications in the period January–June 2019, 109 of these pertaining to use/supply/governance of CDs.
Audits of controlled drug registries; structured data in spreadsheet	We audited 6 CDs registers (1231 pages) in ICU, 5 registers (1120 pages) in oncology and 9 registers (1996 pages) in orthopaedics.
ADC transaction logs; structured data in spreadsheet	We received logs of all recorded ICU ADC transactions for the period March–June 2019.

ADC, automated dispensing cabinets; CDs, controlled drugs; ICU, intensive care unit; WOMBAT, Work Observation Method By Activity Timing (a structured data collection tool).

**Table 3:** How an automated dispensing cabinet (ADC) may support or challenge governance of controlled drugs (CDs) in hospital wards

CD governance activity	ADC support	ADC challenge
Safekeeping/Controlling access	Access control by fingerprinting/username and password Double lock drawers (two users/fingerprints to access) No requirement for keys	Not all CDs ‘storable’ in the ADC. Parallel use of ADC and safe and registers required. Access to CD in the fridge only partially controlled, leading to a hybrid system of ADC for storage and paper registers for documentation. Workaround needed to give access to CD in the fridge and alert nurses of the different workflow. Parallel use of ADC by different users, for different transactions, not supported, leading to potential queues to access medications.
Documenting transactions	Documentation synchronous to removal/supply/count of CDs Automatically recorded data for most transactions, through fingerprinting and item selection on screen Automated alerts of potentially incorrect remaining stock balances recorded Users perception of efficiency and completeness of documentation	Reduced flexibility in amending records compared with paper. Returning CDs to the ADC more complex than with safe and registers.
Monitoring	Counts of stock levels blind to documentation of balance Remote monitoring potentially in real-time, through reporting functionalities and remote access to ADC data Users perception of efficiency and greater control	Data recording only partially automated; some data entered by users may not be accurate (eg, reason for discarding a CD), thus affecting monitoring activities.
Reporting and investigating	Easier to identify discrepancies near the time of their occurrence, making it easier to investigate and resolve Investigations involving interviewing staff can be limited to staff that had access to the ADC in the period of interest	Access to CDs in the fridge by unregistered users (while others are still logged in), not detected by the ADC, is possible. Possible risk of illusion of completeness of records.

### Safekeeping and controlling access

Prior to ADC implementation, CDs were kept in a locked safe, with CDs in need of refrigeration kept in an unlocked fridge. CD supply and use were documented in paper registers (‘CD books’).

The ADC was implemented in a new locked medication room created in the ICU, accessible with personal smartcards. Only registered staff members could access the ADC, by fingerprint or individual username and password. CDs were stored in the ADC in drawers which required the fingerprints of two authorised staff for access. However, some CDs could not be stored in the ADC (CDs belonging to patients, those to be discarded and liquid CDs in multidose bottles). Thus, a safe was also placed in the medication room, to store these CDs, leading to parallel use of ADC plus safe for storing CDs. The safe was also needed in case of ADC breakdown.

One CD (clobazam liquid) that needed to be refrigerated was stored in the ADC fridge. The fridge was locked but did not require two fingerprints for opening, thus technically this CD could be accessed by any authorised user accessing the ADC fridge without a witness. Transactions of the CD in the fridge had to be recorded in paper registers. This required a workaround on the ADC that created potential for errors, as shown in a reported incident (Id7). However, this was considered an improvement on previous storage arrangements, as the ADC provided a level of controlled access that was previously absent.

… now is better in the sense that the fridge is locked. [Before the ADC], […] if [the CD] needed to be in a fridge it would just be in the fridge […], there was no real, a way to do it, we’ve got ways to do it now which is exciting. (id4)

Access to the safe required a key, usually held by senior nurses (known locally as ‘access nurses’). However, other nurses held the key when the access nurse was away or busy. Nurses reported finding the keys to the safe as time consuming and delaying access to medications for patients. The ADC removed the need for keys and for the presence of specific staff members—any authorised nurse and witness were sufficient to access CDs in the ADC.

However, the ADC only allowed one transaction at a time, while CDs in the safe could be accessed by multiple nurses at the same time. Some nurses reported having to wait to access medications in the ADC, when in use. However, we observed nurses queuing to access the ADC rarely (online supplemental appendix 1). In addition, it was possible (and observed) for a nurse to access the ADC when a previous user had not ‘exited’ the transaction and therefore to use their login.

10.1136/ejhpharm-2020-002552.supp1 Supplementary data

### Documenting use

Removing (or returning) CDs from (or to) the ADC required entering data in the ADC documenting both transaction and remaining levels of stock. The ADC automatically recorded date, time and staff members involved (through their fingerprints), as well as the patient name, prescriber name and the CD as selected on screen. Additional data entry was required to add any discarded amounts, and remaining levels of stock. In contrast, a CD could be removed from the safe but documentation in the paper register could be delayed and possibly forgotten.

In case of incorrect data entry of remaining stock levels, the ADC alerted the users by inviting a recount, thus contributing to preventing discrepancies due to counting errors.

If you happen to put in like fentanyl 30 and there’s 29 … It actually alerts you. […] previously you would have just read 30 and three days later someone would have picked it up. […] this way, I think, it prevents those kinds of incidents (id6-9)

We found that removing a CD from the ADC (also including, when necessary, preparing the medication for administration) took two nurses between ~1 min (66 s) and ~12 min (748 s); the corresponding task performed with the safe and registry took from ~1.5 min (98 s) to ~8 min (472 s) (table 4). However, nurses perceived removing and documenting CDs from the ADC to be less time consuming than with safe and registers.

**Table 4:** Time on specific tasks with controlled drugs (CDs) in the medication room, using automated dispensing cabinet (ADC) and safe with registers

			Time (s)			
		Number of tasks	Mean	Minimum	Maximum	Median
ADC	Issue	41	360	66	748	331
	Return/Waste	1	108	108	108	108
	Count	1	632	632	632	632
	Other	4	301	195	358	325
Safe	Issue	25	242	98	472	208
	Return/Waste	0	.	.	.	.
	Count	2	483	469	496	483
	Other	2	225	48	401	225
Both ADC and safe	Issue	1	625	625	625	625

The data exclude tasks related to the CD in the fridge. Times presented in this table must be interpreted with caution. Samples are too small to be considered valid. Tasks performed with ADC are not directly comparable to the corresponding tasks performed with the safe and registries. The medications involved were different (unit doses in the ADC, bottles in the safe) and times of preparation may be significantly different for the different types of medication. The numbers of CDs stored in the ADC and safe were different—potentially affecting CD counts.

Compared with paper registers, the ADC restricted flexibility for amending records, such as documenting later discarded amounts, or returning an unused CD to the ADC. A workaround had to be devised for these tasks using the function ‘miscellaneous returns’. We observed how some nurses had difficulties completing these tasks on the ADC, and similarly they reported:

… when you’re taking [a CD], […] if you forget to enter the waste, […] in the book, there will be an empty space and then you might think of it. […] Even with ADC you have to come back sometimes, but sometimes you tend not to. Like it happened to me once, and then it’s a bit tricky […] I don’t know how to do that. (id10-13)

Analysis of the ADC transaction logs showed that for a total of 2632 CDs removed from the ADC, 64% had waste recorded (online supplemental appendix 2—table A) and 81% of these had waste recorded at the time of removing the medication. Comparable data for registers were not available.

10.1136/ejhpharm-2020-002552.supp2 Supplementary data

Nurses and pharmacy staff commented on how documentation in the ADC, compared with handwritten data in registers, was more legible and complete. In our audits of paper ICU CD registers, we found about 5% of doses annotated with asterisks or with some inaccuracies in documented amounts. Comparable data patterns were also found in registers on control wards (online supplemental appendix 2—table B).

### Monitoring

Monitoring for CD losses was done by: daily counts of available stock against documented balances; periodic audits of registers against stock in the safe and generation of ADC reports.

With the ADC, CD counts were ‘blind’, that is, the nurses did not know the recorded balance on the system before counting items in drawers. The opposite was the case for counts of CDs in the safe—the level of stock documented was in view while counting and may have influenced the stock check.

[in CDs counts with the ADC] I feel that people actually check more than just writing the book up… … (id6-9)

Nurses reported daily counts of CDs stock to be faster with the ADC than with the safe and registers: ‘*ridiculously quicker… So that’s really good*’. (*id14-20*)

… [with the books] you’ve got to write out every medication and everything. With [the ADC] you can just bang, bang, bang, fingers, and you go through and it’s done. It’s just so much quicker. (id21)

The ADC allowed pharmacy staff to monitor CDs in the ADC remotely by generating reports. For example, regular reports were generated on discrepancies with the CDs in the ADC. It also enabled monitoring from a distance that the CD counts had been done in the ward as expected.

… we have more real time reports. So every day at 10:00 am [the ADC] will look at the last 24 hours […] and sends the ICU Manager a list of discrepancies generated on the ward for her to monitor. … (id1)

### Reporting and investigating

Discrepancies about CDs in the ADC were identified through daily reports and generally resolved within a day, as confirmed by transaction logs analysis (a range of 30 s to 21 hours). Nurses had to document in the ADC a reason for rectifying a discrepancy, but usually entered a generic ‘error’.

Discrepancies with CDs in the safe tended to become apparent at the time of pharmacy audits or when the ward unexpectedly ran out of a CD otherwise documented as still available. Explaining these discrepancies involved reviewing all records since the bottle had been opened for any errors, and assessing whether the loss could be explained by dead space in syringes. Such losses could cumulate over large numbers of doses. The process was reported to be very time consuming for pharmacists.

In our audits, we found 54 discrepancies (losses or gains) for CDs in the safe, documented in CD registers in the ICU during a 6-month period (online supplemental appendix 2—table C). As staff told us, and our audits show, discrepancies were more frequent for medications in multidose liquids than for single doses.

The ADC could also be used to generate a list of those who had accessed the specific CD involved in a discrepancy, and thus limit investigations to these staff instead of questioning everyone on shift.

## Discussion

**Figure 1:**
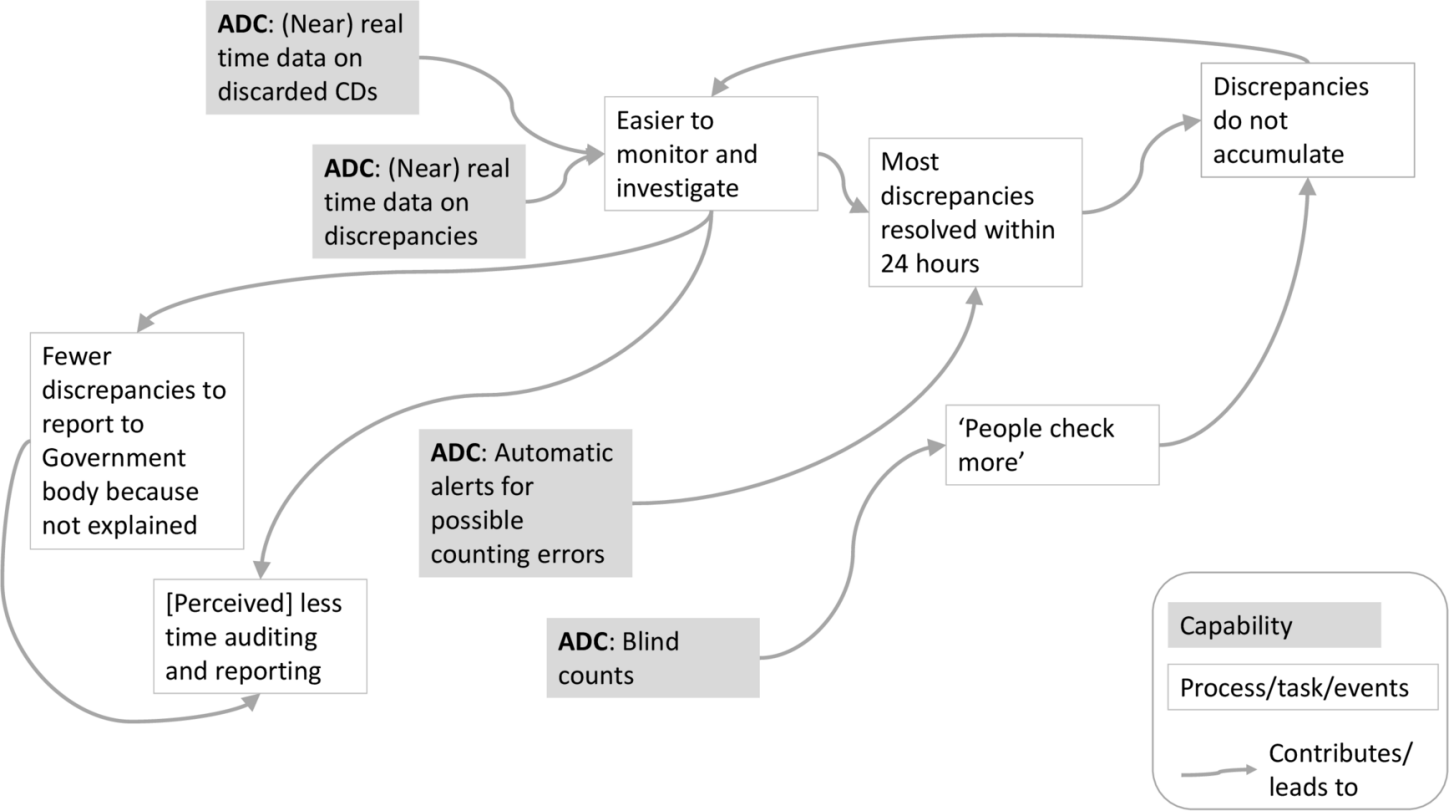
Example of how effects of an automated dispensing cabinet (ADC) on governance of controlled drugs (CDs) may combine. The different effects of using the ADC (such as nurses ‘checking more’ before recording remaining level of CD stock) are related to (or contribute to) other effects (eg, to making discrepancies easier to monitor and investigate).

The ADC affected CD governance tasks in the ICU, through a variety of mechanisms. It afforded concurrent removal (or return) and documentation of CDs; it removed the need to control keys to a safe; afforded automated checks (eg, fingerprints) and electronic interaction with users (eg, questioning counts); and allowed real time, remote access to data (individual transactions and aggregate data) through the generation of reports. Nurses’ counts were ‘blind’ and nurses were found to ‘check more’ when counting remaining level of stock. Pharmacists monitored discrepancies remotely, rather than reviewing registers on the ward (or only doing the latter for CDs in the safe); auditing and monitoring was thus ongoing rather than periodic. These changes, combined, appeared to create positive reinforcing loops (figure 1). However, the ADC only supported governance of CDs in single doses (eg, vials, tablets); a safe and registers were still needed for governance of other CDs. Workarounds were also needed to handle exceptions, such as the single refrigerated drug. Difficulties with the ADCs in relation to multidose liquids (not exclusively CDs) and refrigerated drugs have also been reported elsewhere.^18^

Nurses and pharmacists perceived CD governance to be more efficient with the ADC than with safe and registers. This is consistent with nurses’ perceptions and time and motion studies in other clinical contexts.^10 18 19 27^ We found the question on whether the ADC saves time on CD governance, compared with safe and registers, requires a nuanced answer. The ADC eliminated some time-consuming tasks (eg, transcribing books) but also created work (eg, entering data on screen to resolve discrepancies) and new roles (eg, ADC trainer and ‘super users’). The ADC changed the quality and distribution of tasks, and created new tasks (eg, pharmacists generating reports), as often happens with technology.^28 29^ Time patterns also changed, with monitoring and fixing discrepancies becoming ongoing. The effects extended beyond the ICU, to pharmacy and management dealing with CDs incidents, affecting their use of time. The ADC eliminated frustrating CD governance inefficiencies and this possibly led to perception of saving time.^30^

The ADC made CDs safekeeping and access controls more stringent. In another setting, similar ADC technology was associated with fewer misappropriations of medications.^13^ However, the literature shows that diversion occurs also when ADCs are in place.^3 21^ In the setting we studied, greater quantities than prescribed could theoretically be removed from the ADC, not documented, and the refrigerated CD could be removed by any registered user accessing other medications in the fridge without a witness. Discarded CDs are also a recognised risk for diversion,^3 5^ especially in paediatric settings,^21^ which is not directly affected by use of an ADC or safe.

This study is unique in its focus on CD governance. Our analysis however did not include CD governance in prescribing or administration,^5^ as we did not observe these activities. We did not collect demographic data on the nurses who participated in the interviews; however, from the interviews it was clear that some were new to the ward, while others had senior roles. We were not able to compare the ADC to a baseline pre-implementation. We timed and compared tasks with ADC and safe, but our samples were too small to support significance testing.

## Conclusion

Although creating new challenges, through varied mechanisms, an ADC has the potential to significantly alter CDs governance in clinical areas. But its effects on efficiency and effectiveness seem to be more nuanced and complex beyond merely producing time savings in governance tasks or more stringent controls.

**Key messages**

**What is already known on this subject**

- Effective governance of controlled drugs (CDs) in hospitals is important for policy compliance and for patient and staff safety.
- CD governance through paper-based systems is error-prone and time-consuming.
- Automated dispensing cabinets (ADCs) can be used in hospital wards to store and dispense CDs, and for CD governance tasks.

**What this study adds**

- This study describes the effects of implementing a ward-based ADC on four main CD governance areas: safekeeping/controlling access, documenting use, monitoring and reporting/investigating.
- It highlights a variety of mechanisms through which the use of an ADC both supports and challenges governance of CDs.
- It suggests that an ADC overall improves efficiency of CD governance tasks compared with the use of paper registers, but that these effects are nuanced and complex.

## Data Availability

No data are available. Aggregate data that could be made available have been provided as tables in supplementary files. Our ethics and local approvals preclude making other data, such as interview data, publicly available.
