# Supplementary material for: Automatic dispensing cabinets and governance of controlled drugs: an exploratory study in an intensive care unit
Source: Eur J Hosp Pharm. 2021 May 11;30(1):17–23. doi: 10.1136/ejhpharm-2020-002552 (PMC9811539; doi:10.1136/ejhpharm-2020-002552)
Supplement: Supplementary data [file ejhpharm-2020-002552supp001.pdf]

Appendix 1. WOMBAT data

Summary of data

Data Collection Period - 13 days between 6 June and 24 July

Number of Individual Sessions - 18

Average Duration of Individual Session - 2hours 23mins (Range 1 hour to 4 hours)

Total Hours of Observation - 42 hours 44 mins

Total Tasks Recorded – 686

Inactive Time – 28hours 53mins

Inactive Tasks – 324

Active Time – 15hours 13mins*

Active Tasks - 362

*includes multitasking time (i.e., medication room being used by two or more individuals for separate purposes)

Demand on medication room

There were 56 instances of 'room multitasking' (e.g. multiple nurses working on different medications' tasks).

There were 18 instances where someone was observed to "look in the room and leave" (as if looking for someone or deciding to come back later because the ADC was in use).

There were 9 instances where someone was observed "waiting inside" the medication room (to access the ADC).

Overall time on tasks in medication room

	Time (Seconds)				
	Number of Tasks	Mean	Minimum	Maximum	Median
ADC	207	165	12	1526	78
Safe	29	258	48	496	210
Both ADC & Safe	5	571	177	1282	523
Other*	121	85	5	553	48

*Other includes for example open shelves or EMM.

Time on drug specific tasks in medication room

Times presented in the tables below must be interpreted with caution. Samples are too small to be considered valid. Tasks performed with ADC are not directly comparable to the corresponding tasks performed with the safe and registries. The medications involved were different (unit doses in the ADC, bottles in the safe) and times of preparation may be significantly different for the different types

of medication. The numbers of CDs stored in the ADC and safe were different – potentially affecting CD counts. CD counts always include both ADC and safe, and a 'hybrid' fridge item (stored in the fridge but counted with registries).

Regular drugs

		Regular Drugs				
			Time (Seconds)			
		Number of Tasks	Mean	Minimum	Maximum	Median
ADC	Issue	111	80	17	366	61
	Return/Waste	28	52	12	284	42
	Count	0				
	Other	14	335	47	1526	91

Controlled drugs (overall, excluding item in the Fridge)

		Controlled Drug				
			Time (Seconds)			
		Number of Tasks	Mean	Minimum	Maximum	Median
ADC	Issue	41	360	66	748	331
	Return/Waste	1	108	108	108	108
	Count	1	632	632	632	632
	Other	4	301	195	358	325
Safe	Issue	25	242	98	472	208
	Return/Waste	0	.	.	.	.
	Count	2	483	469	496	483
	Other	2	225	48	401	225
Both ADC & Safe	Issue	1	625	625	625	625

Tasks with both a regular and a controlled drug

		Both Regular and Controlled Drugs				
			Time (Seconds)			
		Number of Tasks	Mean	Minimum	Maximum	Median
ADC	Issue	7	349	72	636	300
	Return/Waste	0				
	Count	0				
	Other	0				
Safe	Issue	0				
	Return/Waste	0				
	Count	0				
	Other	0				
Both ADC & Safe	Issue	4	557	177	1282	385

Controlled drugs – one drug vs multiple drugs

Where the task involves removal of one drug (with no other transactions performed):

		One Drug & Single Transaction				
			Time (Seconds)			
		Number of Tasks	Mean	Minimum	Maximum	Median
ADC	Issue	2	148	111	184	148
Safe	Issue	6	190	98	244	202

Where the task involves removal of one drug, with additional tasks also performed (e.g. recording waste or preparing the drug):

		One Drug & Multiple Transactions				
			Time (Seconds)			
		Number of Tasks	Mean	Minimum	Maximum	Median
ADC	Issue	33	348	66	748	301
Safe	Issue	16	227	119	472	201

When the task involves removal of two or more drugs, with additional tasks also performed:

		**Two or More Drugs & Multiple Transactions**				
			**Time (Seconds)**			
		**Number of Tasks**	**Mean**	**Minimum**	**Maximum**	**Median**
ADC	Issue	6	496	341	679	486
Safe	Issue	3	431	381	461	451
