# Supplementary material for: Automatic dispensing cabinets and governance of controlled drugs: an exploratory study in an intensive care unit
Source: Eur J Hosp Pharm. 2021 May 11;30(1):17–23. doi: 10.1136/ejhpharm-2020-002552 (PMC9811539; doi:10.1136/ejhpharm-2020-002552)
Supplement: Supplementary data [file ejhpharm-2020-002552supp002.pdf]

Appendix 2 – Additional tables

Analysis of ADC transaction logs

Table A – Controlled Drugs – Time from removing a dose from ADC ('issue') to documenting waste (ADC transaction logs, 26 March – 30 June)

Drug (Controlled)	Issue Transactions	Waste Transactions		Waste Concurrent with Issue		Waste by Separate Transaction to Issue								
	n	n	% of Issued	n	% of Total Waste	n	% of Total Waste	Average Time	Range Min	Range Max	<10m	10m - 1hr	1hr - 24hr	>24 hr
Bupivacaine + Fentanyl	9	3	33.3%	3	100.0%	0	n/a	n/a	n/a	n/a				
Clobazam	150	52	34.7%	46	88.5%	6	11.5%	0:29:06	0:00:30	2:48:54	5		1	
Clonazepam	2	2	100.0%	1	50.0%	1	50.0%	0:01:57	0:01:57	0:01:57	1			
Diazepam	156	57	36.5%	48	84.2%	9	15.8%	0:22:46	0:00:20	3:18:01	8		1	
Fentanyl	640	390	60.9%	326	83.6%	64	16.4%	2:37:09	0:00:17	84:12:10	44	4	15	1
Ketamine	229	194	84.7%	110	56.7%	84	43.3%	4:17:27	0:00:23	84:12:55	10	10	63	1
Midazolam	765	593	77.5%	495	83.5%	98	16.5%	0:55:14	0:00:15	9:25:36	56	16	26	
Morphine Hydrochloride	75	49	65.3%	44	89.8%	5	10.2%	0:00:36	0:00:14	0:00:54	5			
Morphine Sulfate	325	237	72.9%	195	82.3%	42	17.7%	4:28:15	0:00:19	172:00:21	37	1	3	1
Morphine Sulfate MR	57	49	86.0%	43	87.8%	6	12.2%	0:01:30	0:00:18	0:06:27	6			
Oxycodone	32	25	78.1%	24	96.0%	1	4.0%	0:00:31	0:00:31	0:00:31	1			
Phenobarbital	31	17	54.8%	15	88.2%	2	11.8%	0:02:33	0:01:01	0:04:05	2			
Phenobarbitone	1	0	0.0%	0	n/a	n/a	n/a	n/a	n/a	n/a				
Propofol	157	12	7.6%	2	16.7%	10	83.3%	6:25:49	0:55:18	16:03:49		2	8	
Thiopentone	3	1	33.3%	1	100.0%	0	n/a	n/a	n/a	n/a				
Total *	2632	1681	63.9%	1353	80.5%	328	19.5%	2:40:26	0:00:14	172:00:21	175	33	117	3

* Not including the controlled drug in the fridge – accessed by generic 'fridge item' in the ADC and documented in registries

Analysis of data from audits of registries of controlled drugs

Table B. Frequency of documentation inaccuracies in controlled drugs registries in the ICU and control wards (January – June 2019)

**Type of documentation inaccuracy**	**ICU n (%)**	**Oncology n (%)**	**Orthopaedics n (%)**
	(doses*: 5783)	(doses*:2930)	(doses*:3750)
**Asterisks** representing corrections to the register	305 (5.3%)	132 (4.5%)	282 (7.5%)
**Calculation errors** identified by nurses or pharmacists	9 (0.2%)	3 (0.1%)	48 (1.3%)
**Other** (e.g. balance changed incorrectly)	3 (0.1%)	1 (0.0%)	9 (0.2%)
**Missing amounts** (given, discarded, balance)	47 (0.8%)	26 (0.9%)	20 (0.5%)

% are calculated on number of doses. * The number of doses is the number of entries documented in registries – i.e. number of times medications have been recorded as given (or not) to patients. The table does not include data about discrepancies, reported in a separate table.

Table C. CD discrepancies (losses and gains) in the ICU and control wards in the study period (January – June 2019)

	**ICU (*) [pre/post ADC] n (%)**	**Oncology [no ADC in use] n (%)**	**Orthopaedics [no ADC in use] n (%)**
	(doses*: 5783)	(doses*:2930)	(doses*:3750)
Discrepancies identified by nurses/pharmacists	54 (0.9%)	11 (0.4%)	37 (1.0%)

(*) Limited to discrepancies identified in registries (excluding discrepancies in ADC). % are calculated on number of doses.
* The number of doses is the number of entries documented in registries – i.e. number of times medications have been recorded as given (or not) to patients.

Comparative analysis of data from audits of controlled drugs registries and ADC transaction logs

Table D. CD discrepancies (losses and gains) in the ICU and control wards in the three months before and after ADC implementation (as recorded in registries and ADC transactions logs)

	**Jan-March 2019 (pre-ADC) ***			**April – June 2019 (post-ADC)**		
	**n of doses**	**Losses N (%)**	**Gains N (%)**	**n of doses**	**Losses N (%)**	**Gains N (%)**
ICU						
Medications in bottles	1597	15 (0.94%)	1 (0.06%)	2273	30 (1.32%)	6 (0.26%)
Medications in unit doses	1792	2 (0.40%)	0	121 [in registries]	0	0
				7366 (*) [in ADC]	15	14
**Oncology** [no ADC in use]						
Medications in bottles	336	4 (1.19%)	0	409	5 (1.22%)	2 (0.49%)
Medications in unit doses	1045	0	0	1140	0	0
**Orthopaedics** [no ADC in use]						
Medications in bottles	1123	19 (1.42%)	1 (0.09%)	1031	14 (1.36%)	2 (0.19%)
Medications in unit doses	889	0	0	707	0	0
